# Circadian Light Hygiene Is Associated with Anemia Markers in Young Adults

**DOI:** 10.3390/biology14121649

**Published:** 2025-11-23

**Authors:** Denis Gubin, Julia Boldyreva, Sergey Kolomeichuk, Oliver Stefani, Aislu Shigabaeva, Larisa Alkhimova, Marina Tchaikovkaya, Dietmar Weinert, Germaine Cornelissen

**Affiliations:** 1 Laboratory for Chronobiology and Chronomedicine, Research Institute of Biomedicine and Biomedical Technologies, Tyumen State Medical University, 625023 Tyumen, Russia; h_aislu@mail.ru; 2 Department of Biology, Tyumen State Medical University, 625023 Tyumen, Russia; tgma.06@mail.ru; 3 Tyumen Cardiology Research Center, Tomsk National Research Medical Center, Russian Academy of Science, 625026 Tyumen, Russia; 4 Laboratory for Genomics, Metabolomics and Proteomics, Research Institute of Biomedicine and Biomedical Technologies, Tyumen State Medical University, 625023 Tyumen, Russia; sergey_kolomeychuk@rambler.ru; 5 Laboratory of Genetics, Institute of Biology, Karelian Research Centre, Russian Academy of Sciences, 185910 Petrozavodsk, Russia; 6 Department Engineering and Architecture, Lucerne University of Applied Sciences and Arts, 6048 Horw, Switzerland; oliver.stefani@hslu.ch; 7 School of Natural Sciences, University of Tyumen, 625003 Tyumen, Russia; l.e.alkhim@gmail.com; 8 Department of Nursing, Tyumen State Medical University, 625023 Tyumen, Russia; varan13@mail.ru; 9 Department of Zoology, Institute of Biology, Martin-Luther-University Halle-Wittenberg, 06120 Halle, Germany; dietmar.weinert@zoologie.uni-halle.de; 10 Department of Integrative Biology and Physiology, University of Minnesota, Minneapolis, MN 55455, USA; corne001@umn.edu

**Keywords:** hemoglobin, mean corpuscular hemoglobin (MCH), anemia, circadian, light hygiene, actigraphy, blue light exposure

## Abstract

Background: Light exposure (LE) and its influence on circadian rhythms are recognized to impact various physiological domains, yet their specific associations with hematological status, particularly in high-latitude environments, remain underexplored. Understanding these links could offer insights into maintaining hematological health. Methods: This study investigated the relationship between 24 h light exposure (LE), blue light exposure (BLE), sleep, and physical activity (PA) and hematological markers in 85 young adults (18–25 years). Participants underwent simultaneous 7-day monitoring using actigraphy and RGB sensors, coupled with morning blood sampling for hemoglobin (HGB), hematocrit (HCT), mean corpuscular hemoglobin (MCH), and red blood cell distribution width (RDW-CV). Results: Univariate analyses revealed significant correlations: normalized BLE amplitude (NA BLE) positively correlated with HGB (r = 0.369, *p* = 0.001) and MCH (r = 0.378, *p* < 0.001). A later BLE acrophase correlated with lower HGB and MCH, but higher RDW-CV. Later PA acrophase was associated with lower MCH and higher RDW-CV, while later bedtime correlated with lower HGB and MCH. Multivariate regressions confirmed that a larger NA BLE predicted higher HGB (β = 0.206, *p* = 0.037) and MCH (β = 0.377, *p* < 0.001), and an earlier BLE acrophase predicted higher MCH and smaller RDW-CV. Conclusions: Advantageous circadian patterns of BLE and PA are linked to a favorable hematological status in young adults during the light-deficient fall season at higher latitudes, underscoring the importance of optimizing light and activity timing for hematological health.

## Simple Summary

This study investigated how circadian light exposure (LE), particularly blue light exposure (BLE), and activity timing relate to hematological variables in young adults residing at high latitudes. Eighty-five medical students wore actigraphs with light sensors for seven days, measuring LE, BLE, and physical activity (PA). Morning blood samples assessed hemoglobin (HGB), hematocrit (HCT), mean corpuscular hemoglobin (MCH), and red blood cell size variability (RDW-CV). Key findings showed that larger normalized amplitude of BLE (NA BLE) positively correlated with higher HGB and MCH. An earlier BLE acrophase (peak timing) was linked to higher MCH and smaller RDW-CV, indicating more uniform red blood cell size. Conversely, later acrophases of BLE and PA were associated with lower MCH, larger RDW-CV, and lower HGB, suggesting poorer hematological profiles in this population at higher risk of anemia. Later bedtimes also corresponded to lower HGB and MCH. These associations remained significant after adjusting for sex and age, although males generally had higher HGB and light exposure patterns of earlier phase and larger amplitude. Circadian profiles of BLE and PA were significantly correlated with hematopoiesis-related blood indices. Notably, beneficial marker profiles were linked to an advanced circadian phase and larger light exposure amplitude.

## 1. Introduction

Anemia remains a significant global health challenge, with wide-ranging impacts on morbidity and quality of life [1]. Hematological variables such as hemoglobin (HGB), hematocrit (HCT), and mean corpuscular hemoglobin (MCH) serve as critical biomarkers for assessing erythropoietic function and oxygen transport capacity. While aging, lifestyle factors, and environmental cues—including sleep patterns and light exposure—are well recognized to influence multiple physiological domains such as mood regulation, cognitive performance, metabolism, eating behavior, and immune function, there is a notable paucity of research investigating the relationship between light exposure, circadian light hygiene, and hematological variables or anemia-related markers. Circadian light hygiene can be defined as daily light exposure patterns, characterized by sufficient dynamic range and regularity to support circadian entrainment and prevent adverse health outcomes, often requiring strategic management in modern society [2,3,4]. Recent investigations underscore light’s multifaceted influence on health, encompassing metabolic regulation and circadian integrity. Specifically, light impacts mammalian metabolism independently of circadian rhythms through retinal and hypothalamic pathways, affecting glucose homeostasis and thermogenesis [5,6], with aberrant exposure potentially contributing to metabolic diseases. Light’s therapeutic applications include photobiomodulation for treating illnesses and modulating inflammation, potentially influencing micronutrient deficiencies implicated in anemia [3,7]. The effects of light, both positive and negative, on health are mediated through visual and non-visual systems, impacting circadian disruption and immune modulation, with outcomes contingent on individual age and physiological status [2,3,4,8]. The pervasive nature of anemia, impacting approximately 25% of the global population [9], emphasizes its critical public health relevance. While recent data from the US reveals notable sex-related disparities, with prevalence at 13.0% in females compared to 5.5% in males [10], US prevalence is generally lower than global averages and also lower than in Russia, where sex-related differences are also observed [11]. Anemia prevalence frequently diverges from iron deficiency trends [12], implicating inflammation and other micronutrient deficiencies as key contributors to hemoglobin variability, potentially modulated by circadian disruptions from light exposure. Circadian rhythms, primarily entrained by light, orchestrate numerous biological processes, yet their potential role in modulating erythropoiesis and red blood cell indices remains underexplored in human populations. Circadian regulation of hematopoiesis has been demonstrated in animal models, where hematopoietic stem cells exhibit oscillatory release influenced by sympathetic nervous system innervation [13,14]. Intriguingly, human red blood cells (RBCs), despite lacking nuclei and transcriptional machinery, exhibit cell-autonomous circadian oscillations sustained by non-transcriptional mechanisms such as peroxiredoxin redox cycles [15]. Recent advances have further elucidated RBC circadian physiology; for example, Beale et al. [16] employed dielectrophoresis to characterize daily rhythms in RBC membrane electrophysiology, while Beale et al. [17] developed a novel biochemical assay revealing daily hemoglobin oxidation rhythms in RBCs that correlate with core body temperature cycles and nitric oxide signaling, suggesting a functional role for RBC circadian rhythms in thermoregulation. These findings underscore the physiological relevance of circadian timing mechanisms within erythrocytes and support the hypothesis that circadian light exposure could influence hematopoietic variables. To address this gap, the present study examined associations between objectively measured circadian light exposure—quantified by seven-day actigraphy with blue light exposure (BLE) metrics—and hematological variables in a cohort of 85 healthy young adults. Participants were recruited during a single autumn month in Tyumen, Russia, and provided a morning blood sample for complete blood count analyses. Physical activity and sleep duration were also analyzed using actigraphy to explore their potential relationship with anemia. The inclusion of these factors was also motivated by their impact on general health and the recognition of their role in the pathogenesis of various conditions. Notably, studies have indicated a U-shaped association between sleep duration and anemia risk [18], and a dose-dependent decrease in depression risk with higher physical activity [19].

## 2. Materials and Methods

### 2.1. Study Participants

Eighty-five young adult medical students from Tyumen, Russia (mean age ± SD: 19.30 ± 1.51 years; 23 men; 62 women), provided seven-day actigraphy data during the same autumn month. Participants were screened upon admission, and those with serious chronic or acute diseases were not included. Additionally, individuals engaged in shift work, engaged in night work, or who had crossed more than two time zones within the month prior to the study were excluded. Sample size was determined via convenience sampling from the accessible student population, utilizing available actigraphs simultaneously to minimize bias from environmental changes in LE/BLE and ambient temperature. No formal a priori power calculation was performed, as this was an exploratory study; however, post hoc analyses confirmed adequate power (>80%) for the detected associations.

### 2.2. Actigraphy

Actigraphy data were collected using the ActTrust 2 device (Condor Instruments, São Paulo, Brazil), worn on the non-dominant wrist for seven consecutive days between October 24 and 20 November 2023. Measurements included motor activity (Proportional Integrative Mode, PIM), wrist skin temperature, light intensity, and blue light intensity, recorded at one-minute intervals. Validation studies confirm PIM’s high correlation (r > 0.85) with polysomnography for sleep–wake estimation [20], and its RGB sensor for blue light aligns [21] with CIE S 026:2018 standards for melanopic irradiance [22]. Parameters calculated for physical activity (PA), light exposure (LE), and blue light exposure (BLE) included the MESOR (midline-estimating statistic of rhythm), and 24 h amplitude and acrophase (timing of overall high values recurring each day). Non-parametric indices for PA and BLE included the most active/exposed 10 h period (M10) and the least active/exposed 5 h period (L5) and the time of their onset, as well as the inter-daily stability (consistency across days), intra-daily variability (within-day fragmentation), and relative amplitude (RA), which was calculated as (M10 − L5)/(M10 + L5) for both PA and BLE. Sleep parameters, such as bedtime, wake time, total sleep time, sleep efficiency, and wake after sleep onset (WASO), were also derived using the ActStudio software Version 1.0.26 based on established algorithms. BLE is irradiance (µW·cm^−2^) in the short-wavelength range as measured by the Blue channel of the Condor AcTrust2. ActStudio provides parametric estimates for all measured variables, including LE and BLE. For LE and BLE, these estimates include the MESOR, representing the average 24 h value for the fitted cosine curve; the amplitude, quantifying the extent of daily variation; and the acrophase, indicating the timing of the peak. The normalized amplitude of blue light exposure (NA BLE) is a further informative index, calculated as the ratio of the 24 h amplitude of the fitted cosine curve for BLE data to its MESOR. This normalization allows for standardized comparison of BLE patterns across individuals and was proven to be an informative index of circadian light hygiene in the previous studies [2,3,4,23]. This metric standardizes the dynamic range of blue light exposure relative to its average value, accounting for individual differences and thereby minimizing inter-individual variability.

### 2.3. Complete Blood Count

Fasting blood samples were collected just before actigraphy recordings. A complete blood count (CBC) was performed, which included the determination of the absolute and relative numbers of red blood cell count (RBC) and associated variables: HGB, HCT, MCH, mean corpuscular volume (MCV), mean corpuscular hemoglobin concentration (MCHC), and red cell distribution width—coefficient of variability (RDW-CV). Blood collection was performed in the morning between 8:00 and 9:00 in the University laboratory. Prior to sampling, the venipuncture site (antecubital fossa) was disinfected with two alcohol swabs. Blood was drawn by venipuncture into vacuum tubes and allowed to clot at room temperature for one minute. Samples were centrifuged at 3000 rpm for 10 min, 15 min after collection. The serum was then removed from the clot using a pipette with a disposable tip, transferred to clean tubes, and stored at 4 °C until analysis. Leukocyte populations were differentiated using a Sysmex XN–1000 hematology analyzer, Sysmex Corporation, Kobe (Japan) with adapted reagents, employing fluorescent flow cytometry technology.

### 2.4. Morningness–Eveningness Questionnaire (MEQ)

The Morningness–Eveningness Questionnaire (MEQ) [24], a 19-item self-report tool, was administered to participants to assess chronotype. Scores ranged from 16 to 86, with higher values indicating a morning type and lower values indicating an evening type. The Russian version of the MEQ has been validated [25] and widely used in previous studies, e.g., [26].

### 2.5. Data Analysis

Statistical analyses were performed using STATISTICA 6, Office Libre Calc, and SPSS 23.0. We analyzed associations between actigraphy (LE/BLE metrics) and CBC data using multivariate linear regression. Models were adjusted for sex and age. Potential confounders (PA, chronotype, BMI) were identified a priori and through exploratory analysis, and then stepwise-included if they affected coefficients >10% or improved model fit. The significance threshold for all statistical tests was set at *α* = 0.05. To account for multiple comparisons, the Benjamini–Hochberg False Discovery Rate (FDR) correction was applied, with a critical FDR value of 0.1. Potential multi-collinearity among variables in the models was evaluated using Variance Inflation Factors (VIFs). Post hoc analyses confirmed adequate power (>80%) for the detected associations.

## 3. Results

### 3.1. Study Participants

General demographic and red blood cell hematological characteristics of young adults are described in Table 1. For average 24-h patterns of LE and BLE of the study participants, see Supplementary Figure S1.

### 3.2. Univariate Associations Between Circadian Parameters and Hematological Variables

Table 2 presents linear regression coefficients for associations of circadian parameters (PA, light exposure: LE and BLE), sleep metrics, and chronotype score with hematological variables. Significant associations, adjusted for multiple comparisons using the Benjamini–Hochberg False Discovery Rate (FDR) at a threshold of 0.1, are indicated by an asterisk. Among PA measures, an earlier acrophase was significantly associated with higher MCH (*r* = −0.274, *p* = 0.012) and smaller RDW-CV (*r* = 0.226, *p* = 0.040). An earlier M10 Onset also demonstrated a significant association with a smaller RDW-CV (*r* = 0.243, *p* = 0.027). No other significant associations were observed between PA and hematological variables after correction for multiple testing. BLE parameters showed several significant correlations with hematological variables. Specifically, an earlier BLE acrophase was significantly associated with higher HGB (*r* = −0.322, *p* = 0.003), HCT (*r* = −0.255, *p* = 0.020), and MCH (*r* = −0.272, *p* = 0.013), and a smaller RDW-CV (*r* = 0.291, *p* = 0.008). A larger BLE amplitude and higher MESOR were also significantly associated with higher HGB and HCT. A larger NA of BLE exhibited the most solid associations with higher HGB (*r* = 0.369, *p* = 0.001) and MCH (*r* = 0.378, *p* < 0.001). Sleep timing variables also revealed significant correlations: an earlier bedtime correlated with higher HGB (*r* = −0.225, *p* = 0.041) and MCH (*r* = −0.314, *p* = 0.004). Other sleep parameters, including wake time, total sleep duration, and sleep efficiency, did not show statistically significant associations with hematological variables. The MEQ score indicative of chronotype was also not significantly associated with the hematological variables examined.

**Figure 1:**
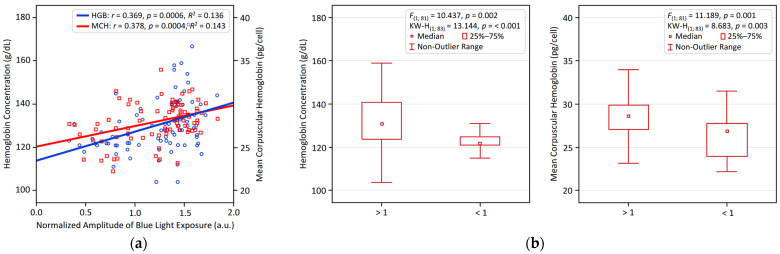
Larger normalized blue light exposure amplitude (NA BLE) is linked to higher hemoglobin and MCH in young adults. (**a**) Linear regression results for NA BLE and hemoglobin and MCH; (**b**) group differences in hemoglobin and MCH comparing large (>1) vs. small (<1) NA BLE.

**Figure 2:**
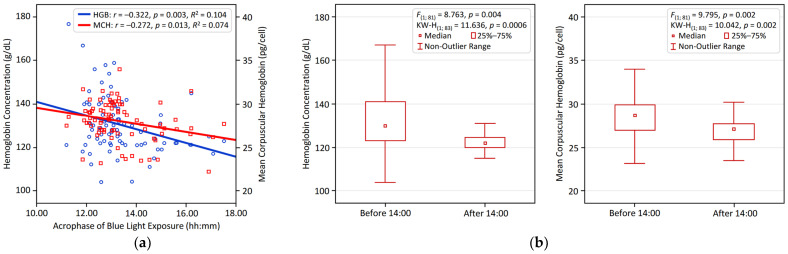
Earlier acrophase of blue light exposure (BLE) is linked to higher hemoglobin and MCH in young adults: (**a**) linear regression results for BLE acrophase vs. HGB and MC; (**b**) group differences in HGB and MCH comparing BLE acrophase before vs. after 14:00.

Figure 1 and Figure 2 visualize the significant associations identified between circadian LE patterns and hematological variables in young adults. Figure 1 illustrates the positive relationship of NA BLE with HGB and MCH, presented through linear regression analyses (Figure 1a) and comparative bar plots depicting participants with NA BLE > 1 vs. < 1 (Figure 1b). Figure 2 shows that an earlier BLE acrophase is significantly linked to higher HGB and MCH, supported by regression analysis (Figure 2a) and comparative bar plots illustrating participants with a BLE acrophase occurring before vs. after 14:00 (Figure 2b).

### 3.3. Multivariate Associations Between Circadian Parameters and Hematological Variables

**Figure 3:**
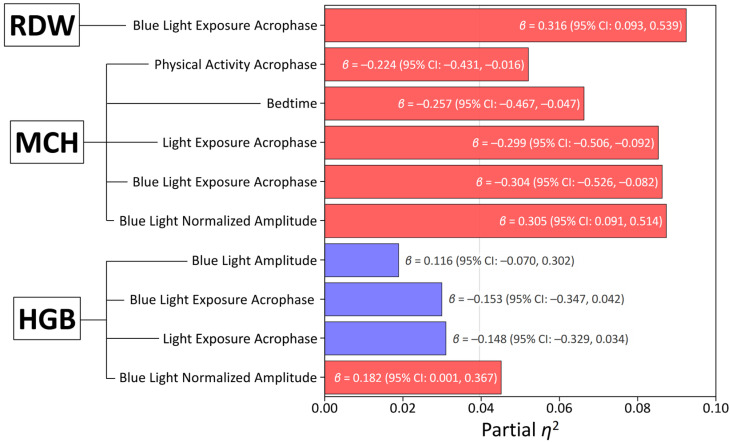
Associations between actigraphy-derived circadian and sleep metrics and hemoglobin. Each predictor was entered into a multivariable regression model individually, with adjustments made sequentially for sex and age. This figure displays standardized beta coefficients (*β*) and their 95% confidence intervals (CIs) for each association. The order of predictors presented reflects their relative contribution as indicated by partial eta-squared (*η*^2^) values. The vertical line denotes the significance threshold (*p* < 0.05). Predictors with statistically significant associations (*p* < 0.05) are indicated by red bars, those in purple were not significant in multivariate model. Abbreviations: RDW, red cell distribution width—CV; MCH, mean corpuscular hemoglobin; HGB, hemoglobin.

Significant circadian predictors, post-correction for multiple testing, were included in a multiple regression model with sex and age as covariates. Whereas no circadian predictors were found for RBC, HCT, MCV, or MCHC, multiple regression analyses, adjusted for sex and age, revealed significant associations of hematological variables with specific circadian light exposure and sleep parameters (Table 3, Figure 3). While the 24 h acrophases of LE and BLE were not significantly associated with HGB after covariate adjustment, this finding reflects the confounding influence of sex, where males exhibited higher HGB (141.5 ± 14.5 vs. 125.7 ± 8.0, *p* < 0.001), superior circadian light hygiene (larger NA BLE: 1.43 ± 0.27 vs. 1.16 ± 0.36, *p* = 0.003), and earlier BLE acrophase (12:48 ± 0:51 vs. 13:39 ± 1:26, *p* = 0.008). Notably, NA BLE still demonstrated a positive association with HGB (*β* = 0.206, 95% CI: 0.012, 0.400, *p* = 0.037, partial *η*^2^ = 0.054), suggesting a subtle independent contribution of 24 h changes in light intensity exposure (Table 3, Figure 3).

Remarkably, several circadian parameters emerged as significant predictors of MCH independently of sex and age, neither of which impacted MCH in the adjusted models (Figure 3). NA BLE was positively associated with MCH (*β* = 0.377, 95% CI: 0.159, 0.595, *p* < 0.001, partial *η*^2^ = 0.130), as was the 24 h acrophase of BLE (*β* = −0.304, 95% CI: −0.526, −0.082, *p* = 0.008, partial *η*^2^ = 0.086). Furthermore, bedtime (*β* = −0.257, 95% CI: −0.467, −0.047, *p* = 0.017, partial *η*^2^ = 0.066) and the 24 h acrophase of PA (*β* = −0.224, 95% CI: −0.431, −0.016, *p* = 0.035, partial *η*^2^ = 0.052) also showed significant positive associations with MCH. An earlier acrophase of BLE was significantly associated with a smaller RDW-CV (*β* = 0.316, 95% CI [0.093, 0.539], *p* = 0.006, partial *η*^2^ = 0.092), indicating greater uniformity in red blood cell size.

Finally, we examined the stability of our main findings by accounting for various co-factors (Supplementary Table S1). Adjusting for physical activity, light exposure mean characteristics, and chronotype did not meaningfully change the associations between the normalized amplitude or timing of BLE and the hematological variables (HGB, MCH, RDW-CV). This suggests that the results are consistent irrespective of mean values of PA, LE, or chronotype score, reflecting the robust predictive power of circadian light hygiene.

## 4. Discussion

This study revealed significant associations between circadian light hygiene and key hematological variables in healthy young adults. A larger BLE NA was independently linked to higher HGB and MCH, while a later BLE acrophase correlated with lower HGB, HCT, and MCH, alongside larger RDW-CV. A later PA acrophase and a later habitual bedtime were also associated with a lower MCH. These findings suggest that circadian light patterns may modulate hematopoiesis, particularly through effects on erythrocytic indices, independently of sex for MCH and RDW-CV. Currently, there is a limited scope of studies characterizing the circadian rhythms of CBC components as evident from a recent review [27], particularly in relation to environmental light cues, which underscores the novelty of our investigation into how circadian light hygiene modulates these variables.

Our previous research at high latitudes found that seasonal changes in circadian light hygiene are associated with changes in morning cortisol, with higher values linked to polar seasons and later timing of physical activity and light exposure [28], altered morning lipids (higher low-density-lipid cholesterol being linked to elevated light at night, higher high-density-lipid cholesterol being linked to earlier timing of light exposure) [28,29,30], and higher clock gene (NR1D1) expression with more abundant light exposure [30]. All these seasonal changes were coupled with a delayed-phase melatonin phase and reduction is its relative amplitude [28]. Notably, the 24 h mean melatonin concentration remained relatively stable despite substantial changes in ambient light patterns [28]. However, since these variables exhibit circadian rhythmicity, single-morning measurements preclude differentiation between associations with overall 24 h means and changes in phase. In this context, it is pertinent that in contrast to HGB and HCT, which demonstrate pronounced 24 h rhythms [27,31], MCH and RDW display minimal 24 h variation. For instance, Sennels et al. [31] reported negligible amplitudes for MCH (0.27% relative amplitude) and RDW (0.16% relative amplitude) in healthy males, with no significant circadian rhythm (*p* > 0.5). Similarly, Hilderink et al. [32] found small within-subject biological variation for MCH (CVI = 0.8%) and RDW (CVI = 0.37%), indicating stability across time points, and no diurnal fluctuations in healthy populations. This result suggests that the observed associations between circadian light exposure and MCH or RDW-CV cannot be attributed to the modulation of their endogenous rhythms, as these variables do not fluctuate significantly over the 24 h day. However, it cannot be excluded that circadian light hygiene may influence the acrophase of HGB and/or HCT, which show pronounced 24 h patterns (e.g., HGB amplitude = 3.28% in [15]). Furthermore, these findings imply that light may exert non-circadian effects on MCH, potentially through pathways independent of any rhythmic modulation, warranting further mechanistic studies. The observed associations between circadian light hygiene and MCH, despite MCH’s minimal intrinsic circadian rhythmicity, suggest a role for indirect photic modulation rather than direct phase-shifting of MCH itself. Light’s potent chronobiotic influence likely optimizes the upstream regulatory mechanisms of erythropoiesis. Specifically, robust light cues, such as a large NA BLE and an earlier BLE acrophase, may entrain circadian rhythms of key metabolic regulators like NR1D1 [30,33], as evidenced by studies showing NR1D1 upregulation in response to bright light exposure [30]. Critically, NR1D1 functions as a heme receptor, directly sensing intracellular heme concentrations to coordinate circadian rhythms with metabolic processes, including those involved in heme synthesis and turnover [34,35]. NR1D1 also can receive feedback from dietary iron [36]. Given that MCH reflects hemoglobin content, and heme is a fundamental component of hemoglobin, NR1D1′s direct interaction with heme positions it as a key modulator of red blood cell maturation. Furthermore, the secondary associations of MCH with bedtime and the acrophase of physical activity, which are themselves strongly modulated by circadian light exposure, support the hypothesis that light patterns indirectly influence MCH by synchronizing broader circadian physiology underpinning hematopoiesis, with NR1D1 acting as a central sensor of metabolic state within this network.

For mechanistic context, it has been previously established that mouse hematopoietic stem cells (HSCs) and their progenitors exhibit robust circadian rhythms, with peak activity occurring approximately 5 h after light onset [13], leading to higher stem cell mobilization. These rhythms are regulated by sympathetic nervous system-mediated noradrenaline secretion, which modulates CXCL12 expression in the bone marrow niche. Importantly, unlike humans, mice are nocturnal and their resting period occurs during the daylight phase when HSC release peaks. This neurally driven circadian release of HSCs may promote stem cell niche regeneration and tissue homeostasis. Our findings suggest that in humans, circadian light patterns could similarly influence hematopoiesis via adrenergic pathways, potentially accounting for the observed associations between BLE and erythrocytic variables such as MCH. Furthermore, the amount of brain iron regulates circadian rhythms by modulating PER1 expression and clock genes like *Clock* and *Bmal1*. Iron deficiency enhances locomotor activity, and iron overload inhibits it [37]. This relation provides further insight into how iron homeostasis may interact with circadian factors in hematopoiesis.

The present findings carry important clinical implications within the broader context of anemia. Anemia, characterized by diminished hemoglobin and impaired oxygen delivery, contributes substantially to the global disease burden. Our study suggests that circadian light hygiene—through its entrainment of endogenous rhythms—may represent a modifiable environmental factor influencing erythropoietic variables. Notably, the exclusive association of blue light exposure metrics with MCH, a key indicator of red blood cell hemoglobin content, underscores a potential mechanistic link between circadian photic input and red blood cell quality. Given the scarcity of prior research in this domain, these results highlight the need to consider circadian light exposure as a novel factor in hematological health. It is particularly relevant for populations at risk of circadian disruption, such as shift workers or individuals residing at extreme latitudes, who may be more susceptible to anemia. In line with this line of thought, Alves et al. [38] demonstrated that poor sleep quality among firefighters, a shift-work profession prone to circadian disruption, correlates negatively with hematological variables (e.g., RBC, HGB, HCT), potentially inhibiting erythropoiesis even within reference ranges. Future longitudinal and interventional studies are essential to elucidate causality and evaluate whether optimizing circadian light hygiene could serve as a non-pharmacological strategy to mitigate anemia risk and improve erythropoietic status. In a related vein, Zhu et al. (2025) [39] found that hemoglobin levels exhibit a nonlinear U-shaped association with respiratory infection risk (lowest risk at mid-range HGB, with elevated risk at both low and high extremes), modified by chronotype, which underscores the broader implications of circadian disruptions for hematological health and infection susceptibility, as highlighted in this discussion on light hygiene’s effects on erythropoiesis. Future research should prioritize longitudinal studies to establish causality. Multi-month prospective cohorts tracking circadian LE/BLE patterns alongside serial CBC components in populations prone to light disruption (e.g., shift workers, high-latitude residents) can reveal long-term effects on erythropoiesis and anemia risk. Specifically timed bright blue-enriched light to optimize BLE may directly test whether improved circadian light hygiene enhances erythrocytic indices and NR1D1-mediated heme regulation. Mechanistic studies incorporating biomarkers like melatonin and ferritin are also warranted to elucidate photic cue–hematopoiesis pathways.

Physical activity exhibits a complex relationship with hematological markers of anemia, potentially enhancing erythropoiesis while risking hemolysis or iron depletion, yet none of the reviewed studies consider the timing of exercise relative to circadian rhythms. Hu and Lin [40] note that exercise can increase hemoglobin and red cell mass via stimulated erythropoiesis, offering benefits for anemia management despite controversial results. Caimi et al. [41] found that trained athletes have improved red blood cell deformability and elevated MCV and MCHC, although a higher VO_2_max correlates with lower MCH and MCHC. Mairbäurl [42] describes “sports anemia” in athletes as dilutional hematocrit decline due to plasma expansion that is offset by an increased total red cell mass and hemolysis favoring younger, more flexible cells. Wouthuyzen-Bakker and van Assen [43] highlight exercise-induced iron deficiency anemia in females from iron losses. Cichoń-Woźniak et al. [44] observed in trained male rowers that baseline ferritin concentrations influence post-exercise iron responses: lower-ferritin participants (<75 µg/L) showed transient iron increases followed by declines, while higher-ferritin participants (>75 µg/L) exhibited only decreases, suggesting that baseline iron stores modulate acute iron regulation without altering hepcidin or interleukin-6. Nam et al. [19] indirectly support the protective role of exercise against depression in anemic individuals. These findings suggest that physical activity modulates erythropoiesis variably, with circadian timing unexplored, potentially interacting with light-entrained hematopoiesis, as our study on circadian light exposure shows.

The strong association of larger NA BLE and earlier BLE acrophase with higher MCH and lower RDW-CV suggests a significant role for robust circadian entrainment in hematopoiesis. Given the minimal 24 h variability of MCH and RDW-CV, these findings likely reflect indirect, long-term influences on erythropoiesis. We propose that pronounced daily fluctuations in light intensity (large NA BLE) and an earlier timing of the BLE peak act as potent chronobiological cues, and favor the circadian clock’s synchronization in the bone marrow. This precise timing may enhance iron homeostasis and promote more uniform erythroid differentiation and hemoglobinization, thereby increasing hemoglobin content per cell (higher MCH) and fostering greater consistency in RBC size (smaller RDW-CV). Our findings suggest that individuals at risk of anemia, particularly those with low daylight exposure and disrupted circadian rhythms (e.g., shift workers), can optimize their circadian light hygiene. Seeking bright blue-enriched light exposure earlier in the day can advance light acrophase and increase normalized amplitude.

Study Strengths: A key strength of this investigation is the study’s homogeneous cohort, with participants examined at the same location in Tyumen, Russia, over a single autumn month, thus minimizing environmental biases from variable light exposure. Additionally, the use of the normalized amplitude (NA) of BLE accounts for inter-individual variability in light exposure, providing a more precise measure of circadian light hygiene and enhancing the reliability of associations with hematological variables. Notably, even when incorporating mean and amplitude of PA, mean LE, or chronotype score as potential confounders, the significant associations between blue light exposure (amplitude and timing) and hematological variables (HGB, MCH, RDW-CV) persisted, indicating that circadian light hygiene exerts an independent influence. To further enhance transparency, we have included a completed JBI Critical Appraisal Checklist for Analytical Cross-Sectional Studies as Supplementary Table S2, affirming the methodological robustness of our study.

Study Limitations: Our study is subject to several limitations. Circadian light hygiene is inherently dependent on the photic environment; thus, our results may not generalize to different seasons or latitudes with markedly divergent daylight patterns. Furthermore, the narrow age range of participants likely contributed to the observed lack of age-specific effects on most variables, limiting generalizability to broader age demographics. Future research should investigate these associations across diverse environmental contexts and age groups to ascertain broader applicability. While our findings link circadian BLE to erythrocytic variables, the absence of reticulocyte counts in routine CBCs precludes direct exploration of erythropoiesis impacts. Future studies should incorporate reticulocyte analysis to investigate direct effects on erythropoiesis, potentially leveraging known circadian human stem cells and iron pathways. Furthermore, as electrophoresis was not performed due to reliance on routine CBC data, deeper analysis of hemoglobin profiles was not possible, though it could offer broader insights into hematological responses in future research. Additionally, the cross-sectional design precludes causal inferences, necessitating longitudinal research to elucidate time-related relationships.

## 5. Conclusions

This research reveals significant associations between circadian light exposure patterns and hematological variables, particularly MCH, HGB, and RDW-CV, in young adults during the light-deficient fall season. Larger NA BLE and earlier BLE acrophase were linked to higher MCH and smaller RDW-CV, and NA BLE was independently associated with higher HGB. These findings underscore the supportive role of circadian light hygiene in maintaining hematological health, providing a foundation for future investigations into light’s impact on hematopoiesis.

## Figures and Tables

**Table 1:** Demographic characteristics and red blood cell characteristics of study participants.

Variable	Mean ± Standard Deviation
Age (years)	19.30 ± 1.51
Body mass index (BMI)	21.76 ± 3.72
Sex (male/female)	23/62
Red blood cells (RBCs) (10^12^/L)	4.68 ± 0.42
Hemoglobin concentration (HGB) (g/dL)	130.3 ± 12.9
Hematocrit (HCT) (%)	40.15 ± 3.52
Mean corpuscular hemoglobin (MCH) (pg/cell)	27.99 ± 2.22
Mean corpuscular volume (MCV) (fL)	85.01 ± 9.87
Mean corpuscular hemoglobin concentration (MCHC) (g/D)	321.9 ± 33.7
Red cell distribution width—CV (RDW-CV) (%)	13.12 ± 1.21

**Table 2:** Correlations between circadian parameters and hematological variables ^1^.

	RBC	HGB	HCT	MCV	MCH	MCHC	RDW-CV
Physical Activity							
MESOR	0.098	−0.024	0.024	−0.081	−0.137	−0.023	0.088
24 h Amplitude	0.190	0.176	0.146	−0.079	0.010	−0.007	0.051
24 h Acrophase	0.088	−0.153	−0.086	−0.069	−0.274 *	−0.024	**0.226**
IV	0.042	0.012	0.039	0.096	−0.063	0.043	0.012
IS	0.111	0.116	0.120	−0.021	0.013	−0.135	−0.104
M10	0.132	0.052	0.055	−0.142	−0.094	0.023	0.076
M10 Onset	0.151	−0.029	0.022	−0.112	−0.190	−0.052	**0.243**
L5	−0.044	−0.197	−0.150	−0.014	−0.180	−0.087	0.048
L5 Onset	0.069	0.005	−0.011	−0.093	−0.058	0.039	−0.051
Relative Amplitude	0.064	0.183	0.138	−0.033	0.129	0.098	−0.024
Light Exposure (LE)/Blue Light Exposure (BLE)							
LE MESOR	0.161	0.189	0.199	0.118	0.030	0.084	−0.049
LE Amplitude	0.128	**0.248**	**0.234**	0.157	0.137	0.084	−0.084
LE Acrophase	−0.071	−0.312 *	**−0.223**	−0.128	−0.280 *	−0.038	0.212
BLE MESOR	0.163	**0.223**	**0.216**	0.121	0.070	0.094	−0.078
BLE Amplitude	0.155	0.279 *	**0.253**	0.143	0.148	0.095	−0.114
BLE Acrophase	−0.093	−0.322 *	**−0.255**	−0.126	−0.272 *	−0.029	0.291 *
BLE M10	0.155	**0.244**	**0.223**	0.122	0.103	0.105	−0.089
BLE M10 Onset	0.029	−0.162	−0.111	**−0.229**	−0.202	−0.001	0.077
BLE L5	0.202	0.069	0.089	−0.041	−0.153	−0.010	0.035
BLE L5 Onset	0.006	−0.202	−0.126	−0.020	**−0.229**	−0.121	0.112
BLE NA	0.047	0.369 *	**0.259**	0.175	0.378 *	0.037	−0.172
Sleep							
Bedtime	0.045	**−0.225**	−0.154	−0.133	−0.314 *	−0.063	0.184
Wake Time	0.107	−0.078	0.011	−0.029	−0.212	−0.030	0.214
Time in Bed	0.058	0.091	0.105	0.043	0.037	0.014	0.056
Total Sleep	0.072	0.108	0.108	0.037	0.044	0.009	0.030
Sleep Efficacy	0.055	0.056	0.004	−0.004	0.017	−0.046	−0.095
WASO	−0.056	−0.056	0.008	0.030	−0.024	0.028	0.138
Chronotype Morningness–Eveningness Questionnaire (MEQ) Score							
MEQ Score	−0.020	0.051	0.010	0.074	0.100	−0.032	−0.047

^1^ Linear regression coefficients are listed. RBC—red blood cell count; HGB—hemoglobin (amount of oxygen-carrying protein in blood); HCT—hematocrit (percentage of blood volume made up by RBCs); MCV—mean corpuscular volume (average size of red blood cells); MCH—mean corpuscular hemoglobin (average amount of hemoglobin per red blood cell); MCHC—mean corpuscular hemoglobin concentration (average concentration of hemoglobin in RBCs); RDW-CV—red cell distribution width—coefficient of variation (variation in red blood cell size). MESOR—midline-estimating statistic of rhythm, a rhythm-adjusted mean; M10—10 h of highest values; L5—5 h of lowest values; IV—intra-daily variability; IS—inter-daily stability; NA BLE—normalized amplitude of blue light exposure; WASO—wake after sleep onset. Significant associations are in bold. Asterisk (*) indicates statistical significance after Benjamini–Hochberg’s correction for multiple comparisons at false detection rate FDR = 0.1.

**Table 3:** Comparative effects of the strongest actigraphy-based predictors, sex, and age on hematological variables.

Dependent Variable	Predictor	*β* (95% CI)	*p*-Value	Partial *η*^2^
**Hemoglobin**	**Sex**	**−0.491 (−0.683, −0.299)**	**<0.001**	**0.247**
	**BLE NA**	**0.206 (0.012, 0.400)**	**0.037**	**0.054**
	Age	0.028 (−0.154, 0.209)	0.763	0.001
**Mean Corpuscular Hemoglobin**	**BLE NA**	**0.377 (0.159, 0.595)**	**<0.001**	**0.130**
	Age	−0.174 (−0.378, 0.032)	0.096	0.035
	Sex	0.060 (−0.156, 0.277)	0.580	0.004
**Red Cell Distribution Width—CV**	**BLE Acrophase**	**0.316 (0.093, 0.539)**	**0.006**	**0.092**
	Age	0.205 (−0.005, 0.416)	0.055	0.046
	Sex	0.023 (−0.245, 0.199)	0.839	<0.001

*β:* standardized beta coefficient from Parameter Estimates; 95% CI: confidence intervals are rounded to 3 decimal places for brevity; *p*-value: from Univariate Tests (*F*-test *p*-values); partial *η*^2^: effect size from Univariate Tests, indicating the proportion of variance accounted for by each factor. Significant predictors are highlighted in bold.

## Data Availability

The datasets generated and analyzed during the current study are not publicly available due to privacy reasons but are available from the corresponding author on reasonable request.

## Supplementary Materials

The following supporting information can be downloaded at: https://www.mdpi.com/article/10.3390/biology14121649/s1. Table S1: Main sex- and age-adjusted circadian light hygiene predictors of hematological variables corrected sequentially for co-factors of physical activity 24 h mean, physical activity 24 h amplitude, mean 24 h light exposure, light exposure M10 LE, and chronotype score (MEQ). Table S2: JBI Critical Appraisal Checklist for Analytical Cross-Sectional Studies. Figure S1: Average 24-h patterns of light (left) and blue light (right) exposure of the study participants.

## Abbreviations

**List of abbreviations**	
BLE	Blue Light Exposure (irradiance in the short-wavelength range, measured by the Blue channel of the Condor AcTrust2 device).
BLE NA	Normalized Amplitude of Blue Light Exposure (ratio of the 24 h amplitude of the fitted cosine curve for BLE data to its MESOR, used to standardize the dynamic range of blue light exposure relative to its average value).
BMI	Body Mass Index.
CBC	Complete Blood Count.
CIE	Commission Internationale de l’Éclairage (International Commission on Illumination, referenced in relation to melanopic irradiance standards).
FDR	False Discovery Rate (a statistical method used to correct for multiple comparisons, with a critical value of 0.1 in the study).
GBD	Global Burden of Disease (a comprehensive assessment of health loss due to diseases, injuries, and risk factors).
HCT	Hematocrit (percentage of blood volume made up of red blood cells).
HGB	Hemoglobin (amount of oxygen-carrying protein in blood).
HSCs	Hematopoietic Stem Cells
IS	Inter-Daily Stability (a non-parametric index measuring consistency of activity or exposure across days).
IV	Intra-Daily Variability (a non-parametric index measuring within-day fragmentation of activity or exposure).
JBI	Joanna Briggs Institute (an organization that provides tools for evidence-based healthcare, including critical appraisal checklists).
L5	Least Active/Exposed 5 h Period (the 5 h of lowest values for physical activity or light exposure).
LE	Light Exposure (general light exposure, measured via actigraphy in lux).
M10	Most Active/Exposed 10 h Period (the 10 h of highest values for physical activity or light exposure).
MCH	Mean Corpuscular Hemoglobin (average amount of hemoglobin per red blood cell).
MCHC	Mean Corpuscular Hemoglobin Concentration (average concentration of hemoglobin in red blood cells).
MCV	Mean Corpuscular Volume (average size of red blood cells).
MEQ	Morningness–Eveningness Questionnaire (a 19-item self-report tool to assess chronotype, with scores ranging from 16 to 86; higher scores indicate morning type).
MESOR	Midline-Estimating Statistic Of Rhythm (a rhythm-adjusted mean).
NR1D1	Nuclear Receptor Subfamily 1 Group D Member 1 (also known as REV-ERB).
PA	Physical Activity.
PIM	Proportional Integrative Mode (a method for estimating motor activity in actigraphy).
RBCs	Red Blood Cells (erythrocytes; also referred to as red blood cell count in 10^12^/L).
RDW-CV	Red Cell Distribution Width—Coefficient of Variation (variation in red blood cell size, expressed as a percentage).
VIFs	Variance Inflation Factors (used to evaluate potential multi-collinearity among variables in regression models).
WASO	Wake After Sleep Onset (time spent awake after initially falling asleep).
**List of terms**	
Acrophase	Timing of the peak in a circadian rhythm.
Amplitude	Magnitude of variation in a circadian rhythm, peak-to-trough difference (e.g., daily light fluctuation).
Chronotype	Individual’s natural preference for morning or evening activity.
Actigraphy	Non-invasive wrist-worn device monitoring movement, sleep, and light exposure for objective data.
