# Supplementary material for: Circadian Light Hygiene Is Associated with Anemia Markers in Young Adults
Source: Biology (Basel). 2025 Nov 23;14(12):1649. doi: 10.3390/biology14121649 (PMC12729606; doi:10.3390/biology14121649)
Supplement: Supplementary file 1 [file biology-14-01649-s001.zip › biology-3960631-supplementary.pdf]

Supplementary Table S1. Main Sex and Age-Adjusted Circadian Light Hygiene Predictors of Hematological Variables corrected sequentially for co-factors of physical activity 24-h mean, physical activity 24-h amplitude, mean 24-h light exposure, light exposure M10 LE, and chronotype score (MEQ)

**Dependent Variable**	**Added Co-factor**	***β* (95% CI)**	**p-value**	**Partial η^2**
**BLE NA for Hemoglobin**	PA 24-h Mean	**0.207 (0.013, 0.401)**	**0.037**	**0.055**
	PA 24-h Amplitude	**0.197 (0.003, 0.391)**	**0.047**	**0.050**
	LE 24-h Mean	**0.210 (0.010, 0.410)**	**0.040**	**0.053**
	LE M10	**0.209 (0.003, 0.415)**	**0.049**	**0.047**
	MEQ	**0.206 (0.011, 0.402)**	**0.039**	**0.054**
**BLE NA for Mean Corpuscular Hemoglobin**	PA 24-h Mean	**0.379 (0.163, 0.595)**	**<0.001**	**0.135**
	PA 24-h Amplitude	**0.379 (0.159, 0.600)**	**<0.001**	**0.131**
	LE 24-h Mean	**0.392 (0.166, 0.617)**	**<0.001**	**0.133**
	LE M10	**0.401 (0.162, 0.640)**	**0.001**	**0.125**
	MEQ	**0.384 (0.165, 0.603)**	**<0.001**	**0.135**
**BLE Acrophase Red Cell Distribution Width – CV**	PA 24-h Mean	**0.313 (0.090, 0.537)**	**0.006**	**0.091**
	PA 24-h Amplitude	**0.313 (0.087, 0.538)**	**0.006**	**0.089**
	LE 24-h Mean	**0.316 (0.090, 0.541)**	**0.007**	**0.090**
	LE M10	**0.318 (0.085, 0.550)**	**0.008**	**0.087**
	MEQ	**0.316 (0.092, 0.540)**	**0.006**	**0.091**

Note: BLE NA – Blue Light Exposure Normalized Amplitude; PA – Physical Activity, LE – light exposure, LE M10 – 10 hours of highest LE; β: Standardized beta coefficient from Parameter Estimates. 95% CI: Confidence intervals are rounded to 3 decimal places for brevity; p-value: from Univariate Tests (F-test p-values). Partial η^2: Effect size from Univariate Tests, indicating the proportion of variance accounted for by each factor.

Supplementary Table S2. JBI Critical Appraisal Checklist for Analytical Cross Sectional Studies

Question	Answer (Yes/No/Unclear)	Comments
1. Were the criteria for inclusion in the sample clearly defined?	Yes	Inclusion criteria: young adult medical students from Tyumen, Russia, age 19.30 ± 1.51 years, screened for chronic diseases, shift work, or recent time zone travel; exclusion of those with serious conditions.
2. Were the study subjects and the setting described in detail?	Yes	Participants: 85 students (23 men, 62 women); setting: University laboratory in Tyumen, Russia, during autumn (October-November 2023); actigraphy data collected over 7 days.
3. Was the exposure measured in a valid and reliable way?	Yes	Circadian parameters (e.g., BLE NA, acrophase) derived from validated actigraphy (ActTrust 2 device) using established algorithms, measurements at 1-min intervals.
4. Were objective, standard criteria used for measurement of the condition?	Yes	Hematological variables measured via standard CBC using Sysmex XN-1000 analyzer with fluorescent flow cytometry; fasting blood samples collected mornings (8:00-9:00).
5. Were confounding factors identified?	Yes	Models adjusted for sex, age, and potential confounders such as motor activity; VIF checked for multicollinearity; additional adjustments for chronotype and PA in sensitivity analyses.
6. Were strategies to deal with confounding factors stated?	Yes	Multiple regression models included covariates; Benjamini-Hochberg FDR correction (q=0.1) for multiple testing; stability analyses accounted for co-factors.
7. Were the outcomes measured in a valid and reliable way?	Yes	Hematological outcomes from validated CBC; actigraphy parameters calculated using ActStudio software with published methods.
8. Was appropriate statistical analysis used?	Yes	Linear and multiple regression; significance at $p<0.05$; FDR correction; effect sizes (partial η^2) reported.

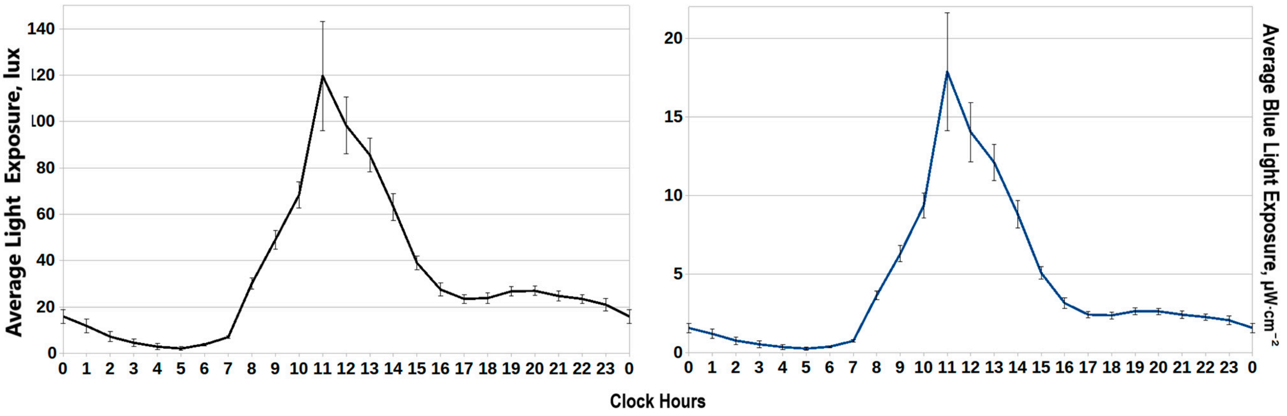

Supplementary Figure S1. Average 24-Hour Patterns of Light (left) and Blue Light (right) Exposure of the Study Participants.
